# Using Machine Learning to Revise the AJCC Staging System for Neuroendocrine Tumors of the Pancreas

**DOI:** 10.3390/cancers17223658

**Published:** 2025-11-14

**Authors:** Jacob Hillman, Quinn Clark, Liam Rehm, Anwar E. Ahmed, Dechang Chen

**Affiliations:** 1 Department of Data and Decision Sciences, Emory University, Atlanta, GA 30322, USA; jhillm6@emory.edu; 2 NYU School of Global Public Health, New York, NY 10003, USA; qwc202@nyu.edu; 3 Sora Schools, Atlanta, GA 30326, USA; liam.rehm@soraschools.com; 4 Department of Preventive Medicine and Biostatistics, Uniformed Services University of the Health Sciences, Bethesda, MD 20814, USA; anwar.ahmed@usuhs.edu

**Keywords:** neuroendocrine tumors of the pancreas, cancer staging, machine learning, EACCD, effect size, dendrogram, C-index, survival curves

## Abstract

**Background**: Staging systems are essential for guiding treatment and predicting outcomes in cancer patients. For pancreatic neuroendocrine tumors, the American Joint Committee on Cancer (AJCC) Tumor, Lymph Node, and Metastasis (TNM) system is the current standard. However, its predictive accuracy is limited, as survival curves often overlap, particularly between Stage I and Stage II. Improved methods of patient stratification are therefore needed. **Methods**: We applied the Ensemble Algorithm for Clustering Cancer Data (EACCD) that involves calculating dissimilarities, ensemble learning, and hierarchical clustering. Data were obtained from the Surveillance, Epidemiology, and End Results (SEER) program of the National Cancer Institute. Models were developed with AJCC TNM variables (T, N, M) and expanded by including patient age. **Results**: The AJCC TNM system achieved a C-index of 0.6656 (95% CI: 0.6473–0.6839), with survival curves showing poor separation. In contrast, the EACCD model using TNM variables produced four prognostic groups with refined and clear separation, yielding a comparable C-index of 0.6685 (95% CI: 0.6518–0.6852). When age was added, EACCD identified five groups with even stronger stratification and a higher C-index of 0.7015 (95% CI: 0.6852–0.7178). **Conclusions**: EACCD provides a refined prognostic framework for pancreatic neuroendocrine tumors, outperforming the AJCC TNM system by offering clearer survival stratification, comparable or higher C-index values, and integration of additional clinical factors.

## Simple Summary

Doctors use cancer staging systems to decide on treatment options and predict how patients may do over time. For pancreatic neuroendocrine tumors, the current staging system does not clearly separate patients into groups with different outcomes, which makes it less reliable for guiding care. In this study, we used a computer-based method called machine learning to analyze patient data and create new groupings. Our approach separated patients more clearly and predicted survival more accurately than the existing system. We also found that including patient age improved predictions even further. These results suggest that updating the staging system with more advanced methods and additional clinical information could provide doctors with better tools for treatment planning and improve the accuracy of patient outcome predictions.

## 1. Introduction

Pancreatic cancer is among the deadliest malignancies in the United States, with 51,980 deaths projected in 2025, exceeded only by lung and colorectal cancers [1]. Although pancreatic neuroendocrine tumors (pNETs) comprise just 1–2% of cases, their incidence has steadily increased, likely due to improved imaging and incidental detection [2]. Compared to pancreatic adenocarcinoma, pNETs occur at a younger median age and have distinct biological and clinical features. They are classified as functional or non-functional, with the latter accounting for the majority and often diagnosed at more advanced stages [3,4]. Accurate staging remains essential for prognostic assessment and guiding treatment decisions, including surgery, targeted therapies, chemotherapy, and somatostatin analogues [5].

The first TNM staging system for pNETs, based on three variables—tumor (T), nodal (N), and metastasis (M)—was introduced by the European Neuroendocrine Tumor Society (ENETS) in 2006, followed by the American Joint Committee on Cancer (AJCC) in its 7th edition in 2010 [6,7]. Comparisons revealed limitations in both systems: ENETS failed to distinguish outcomes between early stages, while AJCC offered poor separation between advanced stages [6,8]. Although the AJCC 9th edition now serves as the current standard [9], limited attempts have been made to refine the AJCC staging systems. Nomogram-based models have been proposed [10] to improve the AJCC 7th and 8th staging systems. However, nomograms rely on proportional hazards assumptions, which are often violated in real-world datasets, restricting their prognostic accuracy.

Machine learning provides an alternative for developing robust staging systems. The Ensemble Algorithm for Clustering Cancer Data (EACCD) [11,12,13] overcomes proportional hazards assumptions by directly identifying prognostic groups through ensemble clustering. EACCD has been successfully applied to multiple malignancies, including breast, thyroid, colorectal, lung, ovarian, endometrial, lymphoma, and melanoma [14,15,16,17,18,19,20,21]. In this study, we apply EACCD to population-based data from the Surveillance, Epidemiology, and End Results (SEER) Program of the National Cancer Institute [22] to refine the AJCC 9th edition staging system for pNETs, addressing the substantial overlap observed between the survival curves of stage I and stage II. We further expand the AJCC system by incorporating age at diagnosis, a key prognostic factor [23], to create a more accurate and clinically useful staging framework.

## 2. Materials and Methods

### 2.1. Data

Data on pNETs were obtained from the SEER Program [22], November 2024 submission, which includes cases diagnosed through 2022. For this study, we restricted analysis to cases diagnosed between 2010 and 2017. The lower bound (2010) was chosen because pancreatic neuroendocrine tumor cases diagnosed before 2010 were grouped with pancreatic exocrine tumors for staging purposes, leading to inconsistent variable definitions. The upper bound (2017) was selected to allow a minimum of 5 years of follow-up for all included cases.

Eligible cases were identified using International Classification of Diseases for Oncology, 3rd Edition (ICD-O-3) site and histology codes. Specifically, we required {Primary Site = Pancreas (ICD-O-3/WHO 2008)} and {Histologic Type ICD-O-3 = 8150–8153, 8155, 8156, 8158, 8240, 8249}, consistent with AJCC 8th edition definitions for pNETs.

From the SEER Program, we extracted information on survival time, vital status, tumor (T), nodal (N), metastasis (M) categories, and age (A) at diagnosis. Following Liu et al. [23], age A was dichotomized into two groups: <50 years and ≥50 years, reflecting its prognostic relevance in pNETs. Levels of T, N, M, and A used in the analysis are summarized in Table 1. For convenience, patients were represented by combinations of variable levels, such as T2N0M0 for patients with T2, N0, and M0. Other clinically relevant variables, such as mitotic count and Ki-67 index, are not captured in the SEER Program and therefore could not be incorporated into this analysis.

**Figure 1:**
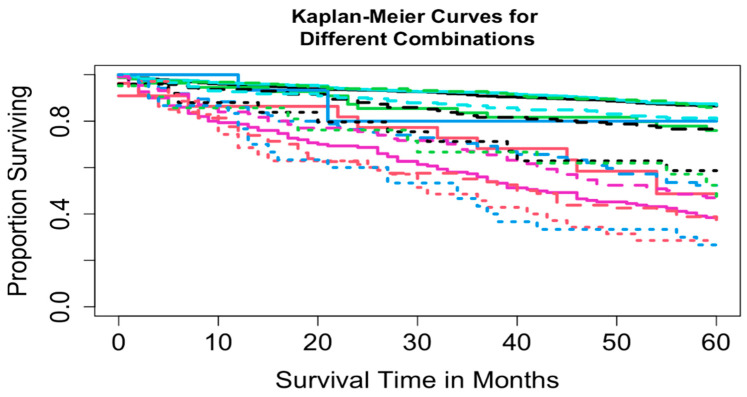
Overall survival curves from 16 combinations for Dataset 1.

**Figure 2:**
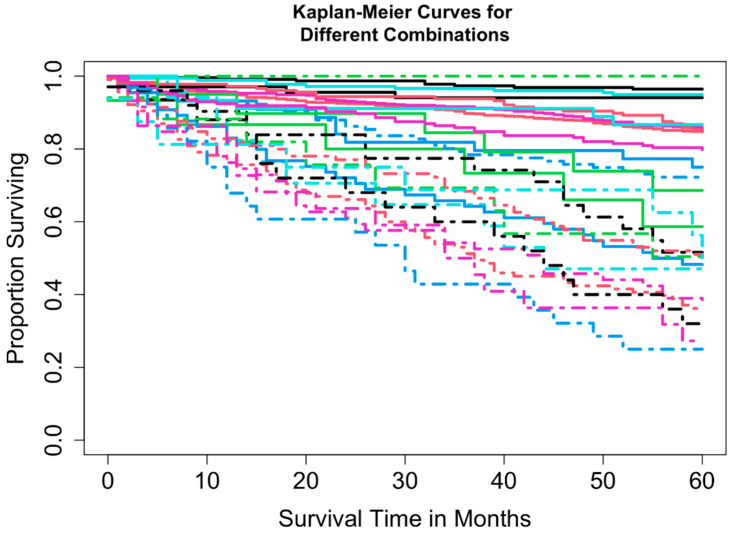
Overall survival curves from 24 combinations for Dataset 2.

Two datasets were constructed: Dataset 1 based on combinations of T, N, and M, and Dataset 2 based on combinations of T, N, M, and A. Dataset 1 included complete information on survival time, vital status, T, N, and M, yielding 16 possible combinations (each containing at least 15 patients) and encompassing 3278 patients. A total of 3225 patients had complete information on survival time, vital status, T, N, M, and A, corresponding to 32 possible combinations. For Dataset 2, combinations with fewer than 15 patients were excluded to maintain sufficient statistical power (a reasonable cutoff, though other thresholds could be used). This resulted in the exclusion of 8 combinations totaling 52 patients (T1N0M1A1, T1N1M0A1, T1N1M1A1, T1N1M1A2, T4N0M0A1, T4N0M1A1, T4N1M0A1, and T4N1M1A1), leaving 3169 patients across 24 combinations in T, N, M, and A for Dataset 2. Table 2 summarizes the key baseline characteristics of the data. Figure 1 and Figure 2 show the overall survival curves for Dataset 1 and Dataset 2, respectively, estimated using the Kaplan-Meier method [24]. We used overall survival as the primary outcome variable, consistent with the survival curves reported in the AJCC 9th edition staging system for pNETs [9].

### 2.2. EACCD

The EACCD is a tool that groups patients based on differences in their survival outcomes. It first measures these survival differences, then refines them using data, and finally organizes patients into similar groups. A more technical description is provided below.

Given a collection of combinations $\left\{C_{1},C_{2},...,C_{n}\right\}$ and nonnegative weights $w_{1},w_{2},...,w_{n}$ with $\sum_{k=1}^{n}w_{k}=1$, the EACCD consists of the following three main steps (see the Experiments section of [13]):

Initial dissimilarity definition: Compute the initial dissimilarity $dis_{0}(C_{i},C_{j})$ for any pair $C_{i}$ and $C_{j}$.

Ensemble learning: For each k with 1≤ k≤n, apply the two-phase Partitioning Around Medoids and the initial dissimilarities $dis_{0}(C_{i},C_{j})$ to partition combinations into k clusters. Define $\delta_{k}(i,j)$ as 1 if $C_{i}$ and $C_{j}$ are assigned to different clusters, and 0 otherwise. The learned dissimilarity is then calculated as $dis(C_{i},C_{j})=\sum_{k=1}^{n}w_{k}\delta_{k}(i,j)$.

Hierarchical clustering: Apply hierarchical clustering to group the combinations based on the learned dissimilarities $dis\;(C_{i},C_{j})$.

Each step can employ different methods. In this study, the initial dissimilarity between $C_{i}$ and $C_{j}$ was defined as the survival difference in the two underlying populations, computed using an effect size based on the Gehan–Wilcoxon test statistic [25]. Equal weights were used in the ensemble step for illustration purposes ($w_{k}=1/n$ for k=1, 2,…, n). For hierarchical clustering, the minimax linkage method was selected [26].

The EACCD algorithm produces a dendrogram representing all possible combinations in terms of survival. This visualization helps researchers and clinicians understand the rationale behind patient groupings. Combinations can be partitioned by horizontally cutting the dendrogram at a specified level of dissimilarity. Harrell’s C-index (concordance index) [27] is then computed to assess the predictive accuracy of the resulting prognostic groups. Typically, the C-index increases with a small number of groups and plateaus as more groups are added. The optimal number of prognostic groups (n*) is identified at the “knee” point of the C-index curve.

It is important to note that the EACCD algorithm simply clusters the survival curves of combinations. Because no labels are available for these combinations, EACCD operates in an unsupervised manner. The goal is to form prognostic groups in which survival curves within a group are more similar to each other than to those in other groups. Viewing the problem in this way simplifies the overall concept of partitioning patients with survival data into prognostic groups.

### 2.3. EACCD Prognostic System

The EACCD algorithm was applied to the SEER dataset to generate an optimal number of prognostic groups (n*). Survival curves for these groups were estimated using the Kaplan–Meier method [24]. The resulting framework—referred to as the EACCD prognostic system—consists of four components: the dendrogram, group assignments, Harrell’s concordance index (C-index), and Kaplan–Meier survival curves.

In this system, risk stratification is assessed by examining the separation of Kaplan–Meier curves among prognostic groups, while predictive accuracy is measured using the C-index, which quantifies concordance between predicted and observed survival.

The EACCD prognostic system has been shown to work well across multiple cancer types in previous studies [14,15,16,17,18,19,20,21], and our results demonstrate that it also works well for pancreatic neuroendocrine tumors. By incorporating age into the staging system alongside the traditional tumor, node, and metastasis (T, N, M) variables, EACCD increases the precision of prognostic assessment beyond existing models.

Clinical application of the EACCD system is straightforward. A patient’s values for the relevant variables (say T, N, M, and age) are first determined. Based on these values, the patient is assigned to one of the EACCD-defined prognostic groups. The Kaplan–Meier curve associated with that group then provides an estimate of survival probability, which can support shared decision-making, guide treatment strategies, and help tailor follow-up care.

### 2.4. Software

All statistical analyses were conducted using R (version 4.4.2) within RStudio (version 2023.06.1 + 524) [28]. The primary R packages employed included survival (version 3.8-3), cluster (version 2.1.8), protoclust (version 1.6.4), along with their respective dependencies. The R package compareC (version 1.3.3), which is based on the methodology described in [29], was also utilized.

## 3. Results

This section presents the results of our analysis. We begin with the survival curves for the AJCC TNM staging system using Dataset 1, which includes the variables T, N, and M. We then examine how the EACCD algorithm stratifies patients in Dataset 1 and display the corresponding survival curves. Finally, we assess EACCD on Dataset 2, which incorporates T, N, M, and A, and present the resulting survival curves. For clarity, the various AJCC prognostic stages are denoted as “stages,” while the corresponding EACCD results are denoted as “groups”.

### 3.1. Survival Curves of AJCC TNM Staging on SEER Data

**Figure 3:**
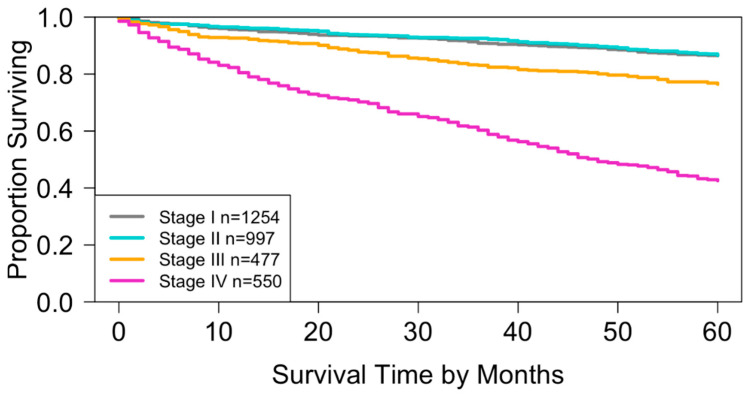
Overall survival for the four stages of the AJCC TNM staging system in Dataset 1. The curves for stage I and stage II show substantial overlap (log-rank test, *p* = 0.4). All other pairwise comparisons indicate significant separation of survival curves (log-rank test, *p* < 0.01).

Using SEER data from this study, the AJCC staging definitions produced four specific stages, as shown in Table 3. Based on this table and Dataset 1, the Kaplan–Meier survival curves in Figure 3 were obtained, and the concordance index (C-index) for the staging system was 0.6656 (95% CI: 0.6473–0.6839).

When compared with the survival curves generated from the National Cancer Database (NCDB) in the AJCC 9th Edition [9], the curves (Figure 3) based on SEER Dataset 1 followed a similar pattern but were slightly lower. Erkaya et al. suggested that this difference may be due to characteristics of the NCDB, which primarily includes patients treated at CoC-accredited hospitals where treatment quality may be higher [30]. Supporting this explanation, Liu et al. reported that overall survival in the NCDB was more than 2% higher than in SEER [31].

### 3.2. EACCD Based Prognostic System in T, N, M

**Figure 4:**
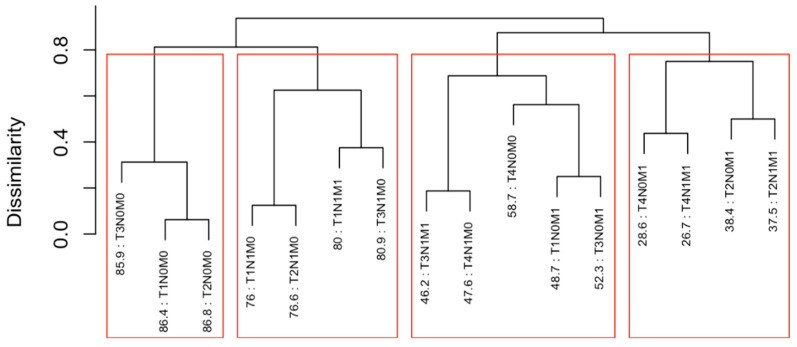
Dendrogram generated by running EACCD on Dataset 1. A 5-year overall survival rate in percentage is given beneath each combination. The dendrogram is cut at n* = 4 (indicated by red rectangles) based on the C-index curve in Figure 5.

**Figure 5:**
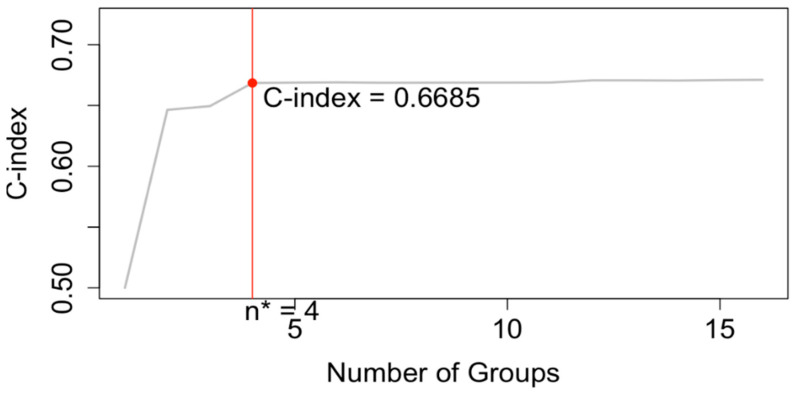
C-index curve based on the dendrogram shown in Figure 4. The dendrogram is cut at n* = 4.

**Figure 6:**
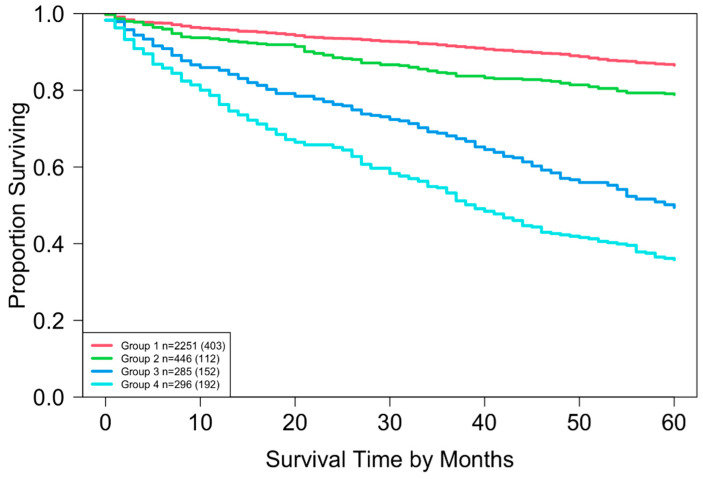
Overall survival of the four prognostic groups in the EACCD TNM system, defined in Figure 4 and summarized in Table 4. The separation between any two adjacent curves is statistically significant (log-rank test, *p* < 0.01). The number in parentheses indicates the total number of deaths.

Running EACCD on Dataset 1 generated the tree-structured dendrogram shown in Figure 4, with the 5-year overall survival rate indicated below each combination. Based on the C-index curve in Figure 5, the dendrogram was cut at n* = 4, resulting in four prognostic groups. These groups, listed in Table 4 as Groups 1 through 4, are ordered by increasing disease severity according to their 5-year overall survival rates. The corresponding survival curves are presented in Figure 6. Conceptually, the EACCD system clusters the 16 survival curves in Figure 1—representing the 16 combinations in Dataset 1—into four distinct groups, whose aggregate survival patterns are shown in Figure 6. The complete system, consisting of the dendrogram (Figure 4), group assignments (Table 4), C-index of 0.6685 (95% CI: 0.6518–0.6852), and survival curves (Figure 6), is referred to as the EACCD TNM system.

### 3.3. EACCD Based Prognostic System in T, N, M, A

**Figure 7:**
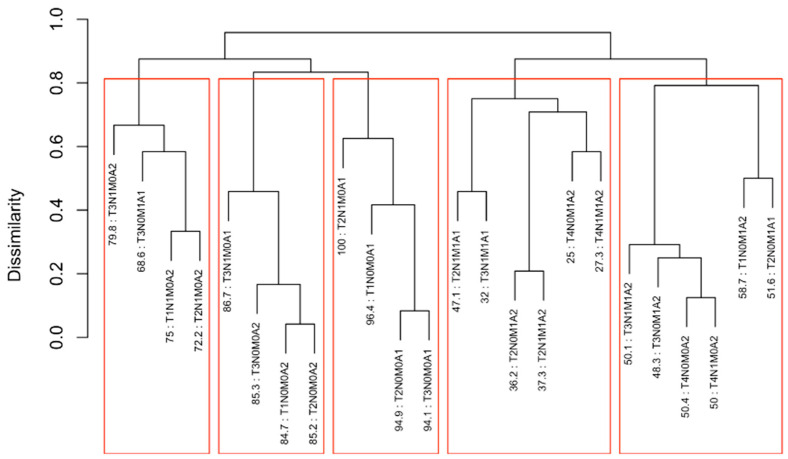
Dendrogram generated by running EACCD on Dataset 2. A 5-year overall survival rate in percentage is given beneath each combination. The dendrogram is cut at n* = 5 (indicated by red rectangles) based on the C-index curve in Figure 8.

**Figure 8:**
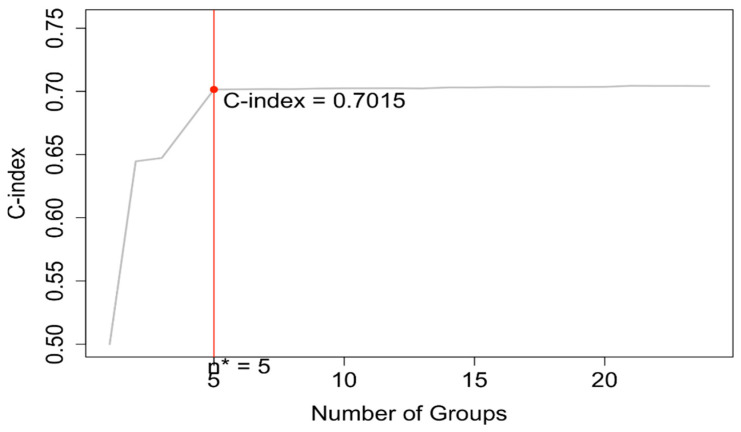
C-index curve based on the dendrogram shown in Figure 7. The dendrogram is cut at n* = 5.

**Figure 9:**
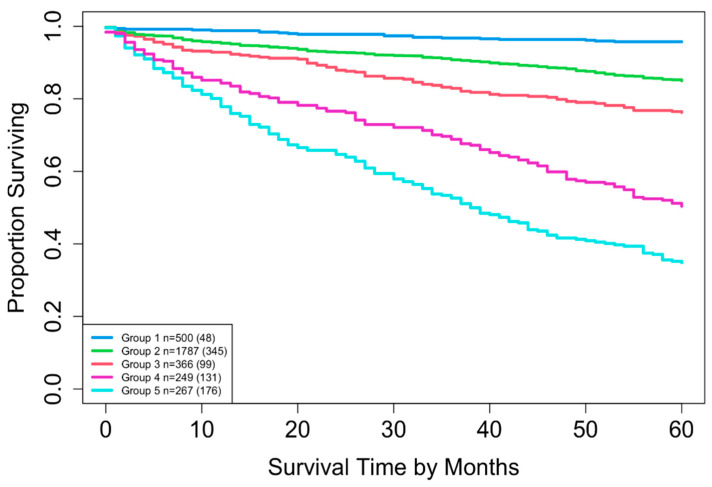
Overall survival of the five prognostic groups in the EACCD TNMA system, defined in Figure 7 and summarized in Table 5. The separation between any two adjacent curves is statistically significant (log-rank test, *p* < 0.01). The number in parentheses indicates the total number of deaths.

EACCD was then applied to expand the AJCC TNM staging system by incorporating variable A. Running EACCD on Dataset 2 generated the dendrogram shown in Figure 7. Based on the C-index curve in Figure 8, the dendrogram was cut at n* = 5, yielding five prognostic groups. Table 5 presents these groups (Group 1 through Group 5) reorganized by increasing disease severity, and their corresponding survival curves are displayed in Figure 9. Geometrically, the EACCD system partitions the 24 survival curves in Figure 2 (corresponding to the 24 combinations in Dataset 2) into 5 groups, with the related survival curves shown in Figure 9. The resulting system, which includes the dendrogram in Figure 7, the assignments in Table 5, the C-index of 0.7015 (95% CI: 0.6852–0.7178), and the survival plot in Figure 9, is referred to as the EACCD TNMA system.

## 4. Discussion

### 4.1. Number of Prognostic Groups

EACCD, as demonstrated above, is a statistical learning algorithm that groups combinations through dendrogram construction. In general, combinations are partitioned into prognostic groups by horizontally cutting the dendrogram at a specified level of dissimilarity. Two extremes arise from this process: one yields a single prognostic group containing all combinations, while the other assigns each combination to its own group. Neither extreme is clinically useful, making the choice of cut point critical. In the EACCD framework, the dendrogram is cut based on the C-index curve. Specifically, the number of prognostic groups is set to n*, determined by the “knee” point of the curve. Typically, the C-index increases rapidly with a small number of groups, then plateaus as additional groups are created. As a result, n* is generally much smaller than the total number of combinations, ensuring simplicity of the system. At the same time, because the “knee” point usually provides a C-index value close to the maximum achievable (corresponding to the maximum number of groups), n* represents an optimal balance between simplicity and predictive performance.

### 4.2. Partitioning Patients by 5-Year Overall Survival Using EACCD

It is informative to explore how EACCD dendrograms divide patients according to their 5-year overall survival. For instance, the dendrogram based on T, N, and M (Figure 4) separates patients into four groups, highlighted by red boxes. The 5-year overall survival ranges for these groups are 26–39%, 46–59%, 76–81%, and above 85%, respectively, demonstrating clear distinctions among the groups. A similar pattern is observed in the dendrogram based on T, N, M, and A (Figure 7), where the cut yields five well-defined groups. Survival at year 5 serves as a snapshot, summarizing the overall prognosis within each group at a specific time point. The survival curves (Figure 6 and Figure 9) further illustrate how patients within each group differ in survival outcomes over time.

### 4.3. Comparison of EACCD TNM with AJCC Staging

The AJCC staging system can be compared with the newly developed EACCD-generated prognostic system in terms of predictive accuracy and risk stratification. As demonstrated earlier, the EACCD TNM system achieved a comparable concordance index (C-index = 0.6685 (95% CI: 0.6518–0.6852)) to that of the AJCC staging system (C-index = 0.6656 (95% CI: 0.6473–0.6839)).

In addition to predictive accuracy, risk stratification was evaluated using Kaplan–Meier survival curves. For the AJCC system, the survival curves of stage I and stage II overlapped throughout the study period (Figure 3), which diminishes the discriminatory ability of the system. Such overlap potentially undermines the prognostic validity of the staging system and may introduce uncertainty in clinical decision-making.

By contrast, the survival curves derived from the EACCD TNM system were well separated across prognostic groups (Figure 6). This clear separation demonstrates that the EACCD system provides more reliable stratification of patients by survival outcomes.

Taken together, these findings indicate that the EACCD TNM system offers comparable predictive accuracy and improved risk stratification compared with the AJCC TNM staging system.

### 4.4. Comparison of EACCD TNMA with EACCD TNM

The EACCD TNMA system extends the EACCD TNM framework by incorporating an additional variable, age, denoted as A. The inclusion of this variable was evaluated in terms of predictive accuracy and risk stratification.

The C-index of the EACCD TNMA system was 0.7015 (95% CI: 0.6852–0.7178), which is significantly higher than the C-index of the EACCD TNM system (0.6685 (95% CI: 0.6518–0.6852)). This finding demonstrates that the addition of age enhances the model’s ability to predict survival outcomes.

Risk stratification was also examined using Kaplan–Meier survival curves. The EACCD TNM system produced four well-separated prognostic groups, whereas the EACCD TNMA system produced five distinct and clearly separated survival curves (Figure 9). The increase in the number of prognostic groups, along with the clear separation between them, indicates that incorporating age allows for finer differentiation among patients with varying survival outcomes.

In summary, the integration of age into the EACCD framework results in the EACCD TNMA system, which provides higher predictive accuracy and more refined risk stratification than the EACCD TNM system.

### 4.5. Clinical Applications of EACCD TNM and TNMA Systems

In clinical practice, the EACCD TNM and TNMA systems can be applied in much the same way as the AJCC staging system or used as a complementary tool alongside the ENETS guidance [32]. Physicians may refer to the survival curves generated by these EACCD-based systems as population-level guides to prognosis, which can help explain general outcomes to patients, compare relative risks across different groups, and facilitate informed decision-making. These curves also play a critical role in treatment planning, for example, by identifying patients at higher risk who may benefit from more intensive or targeted therapies. By providing a robust statistical foundation and practical clinical insights, the EACCD TNM and TNMA systems have the potential to enhance risk stratification, optimize therapeutic decisions, and improve overall patient outcomes.

### 4.6. Effect of Variable Inclusion on EACCD Performance

We have demonstrated two EACCD systems: EACCD TNM, which incorporates the three variables T, N, and M, and EACCD TNMA, which extends the model by adding the variable A. In principle, EACCD is highly flexible and can accommodate any number of prognostic factors or variables. (Continuous variables, however, must be discretized before inclusion.) As the number of variables increases, a larger dataset is generally required to ensure adequate representation of all possible combinations of variable values. The inclusion of additional variables leads to an exponential growth in the number of such combinations, resulting in a denser dendrogram and, typically, a bigger number of prognostic groups. Consequently, the system tends to achieve higher predictive accuracy and finer risk stratification. This trend is evident when comparing the results of EACCD TNM and EACCD TNMA, where the latter exhibits improved prognostic performance due to the inclusion of an additional variable.

### 4.7. Integrating EACCD into Clinical Decision Support

The development of the EACCD prognostic system represents a significant advancement toward personalized medicine in pancreatic cancer. As a data-driven framework, the EACCD system can be continuously refined and expanded through the incorporation of new prognostic factors (e.g., Ki-67, grade), ensuring that the model remains adaptive and relevant within an evolving research landscape. Beyond its analytical strength, the EACCD framework holds great potential for integration into clinical practice through the development of a clinical decision support tool. Such a tool could leverage EACCD algorithms to generate real-time, individualized prognostic groupings for patients, thereby assisting clinicians in tailoring treatment strategies, improving risk assessment, and optimizing patient management. Ultimately, the continued evolution and clinical implementation of the EACCD system could contribute meaningfully to improving survival outcomes and quality of care for patients with pancreatic with pNETs.

### 4.8. Limitations

The EACCD utilizes effect size to define the dissimilarity in survival between two groups. Larger patient numbers yield more accurate effect size estimates [13], which in turn lead to more reliable EACCD prognostic systems. In other words, if all combinations have larger sample sizes, the resulting EACCD system will be more accurate. In this study, the data include about 3000 patients, limiting the sizes of combinations. In Dataset 1, 20% of combinations have no more than 25 patients, and in Dataset 2, 50% of combinations have no more than 25 patients. Such small combinations may not provide reliable effect size estimates and could therefore lead to suboptimal risk stratification. Nevertheless, because the EACCD is data-driven, its accuracy will improve as more patients become available.

Another limitation of this study is the lack of external validation. Validation using independent datasets is essential to confirm the generalizability and robustness of the EACCD TNM and TNMA systems across diverse patient populations. We encourage future studies to perform such validation to support broader clinical application.

## 5. Conclusions

In this study, we applied the machine learning algorithm EACCD to SEER data to develop two prognostic systems for pancreatic neuroendocrine tumors. The EACCD TNM system incorporates three variables—(T)umor, (N)ode, and (M)etastasis—while the EACCD TNMA system further includes (A)ge as a fourth variable. The EACCD TNM system serves as a data-driven revision of the original AJCC TNM staging, and the EACCD TNMA system represents an expansion of the AJCC TNM framework.

Our results demonstrate that the EACCD TNM system provides better patient stratification and higher survival prediction accuracy compared with the AJCC TNM system. Moreover, the EACCD TNMA system further refines patient stratification and achieves improved predictive performance relative to the EACCD TNM system. This, in turn, indicates that the variable age plays an important role in prognostic analysis, as the C-indices of the EACCD TNMA and EACCD TNM systems are 0.7015 (95% CI: 0.6852–0.7178) and 0.6685 (95% CI: 0.6518–0.6852), respectively. These findings highlight the potential of EACCD to generate prognostic groupings that are more precise and clinically informative than traditional staging approaches.

Importantly, the EACCD systems are inherently data-driven and easily updatable, unlike conventional committee-based AJCC staging. In the era of AI and big data in medicine, these models can be rapidly revised to incorporate new measurements, such as emerging clinical, molecular, or genetic biomarkers, thereby continuously improving prognostic accuracy and clinical utility. This adaptability positions EACCD as a flexible and forward-looking tool for precision medicine in pancreatic neuroendocrine tumors.

## Figures and Tables

**Table 1:** Definitions of levels of T, N, M, and A.

Factors	Levels	Definitions
Primary Tumor (T)	T1	Tumor limited to the pancreas, ≤2 cm in greatest dimension
	T2	Tumor limited to the pancreas, >2 cm but ≤4 cm in greatest dimension
	T3	Tumor limited to the pancreas, >4 cm in greatest dimension; or tumor invading the duodenum, ampulla of Vater, or common bile duct
	T4	Tumor invading adjacent organs (stomach, spleen, colon, adrenal gland) or the wall of large vessels (celiac axis, superior mesenteric artery/vein, splenic artery/vein, gastroduodenal artery/vein, portal vein)
Regional Nodes Positive(N)	N0	No regional lymph node involvement
	N1	Regional lymph node involvement
Metastasis(M)	M0	No distant metastasis
	M1	Distant metastases
Age (A)	A1	0 ≤ Age < 50
	A2	50 ≤ Age

**Table 2:** Key baseline characteristics of Dataset 1 and Dataset 2. Percentages at levels of the same variable show the distribution of patients across those levels.

		T				N		M		A	
		T1	T2	T3	T4	N0	N1	M0	M1	A1	A2
Dataset 1		41%	34%	22%	3%	78%	22%	83%	17%		
Dataset 2		41%	34%	22%	3%	79%	21%	84%	16%	20%	80%

**Table 3:** Four stages of AJCC staging in Dataset 1. The number in parentheses indicates the total number of patients.

Stage 1	Stage 2	Stage 3	Stage 4
T1N0M0 (1254)	T2N0M0 (745)	T4N0M0 (25)	T1N0M1 (22)
	T3N0M0 (252)	T1N1M0 (56)	T2N0M1 (150)
		T2N1M0 (142)	T3N0M1 (86)
		T3N1M0 (233)	T4N0M1 (35)
		T4N1M0 (21)	T1N1M1 (15)
			T2N1M1 (81)
			T3N1M1 (131)
			T4N1M1 (30)

**Table 4:** Four patient groups from Figure 4, reorganized in order of increasing disease severity.

Group 1	Group 2	Group 3	Group 4
T1N0M0	T1N1M0	T3N1M1	T4N1M1
T2N0M0	T2N1M0	T4N1M0	T4N0M1
T3N0M0	T1N1M1	T4N0M0	T2N0M1
	T3N1M0	T1N0M1	T2N1M1
		T3N0M1	

**Table 5:** Five patient groups from Figure 7, reorganized in order of increasing disease severity.

Group 1	Group 2	Group 3	Group 4	Group 5
T1N0M0A1	T3N1M0A1	T3N1M0A2	T3N1M1A2	T2N1M1A1
T2N0M0A1	T3N0M0A2	T3N0M1A1	T3N0M1A2	T3N1M1A1
T3N0M0A1	T1N0M0A2	T1N1M0A2	T4N0M0A2	T2N0M1A2
T2N1M0A1	T2N0M0A2	T2N1M0A2	T4N1M0A2	T2N1M1A2
			T1N0M1A2	T4N0M1A2
			T2N0M1A1	T4N1M1A2

## Data Availability

All data files are available from the SEER database at https://seer.cancer.gov/data (accessed on 20 May 2025). A similarly structured source code written by Dr Huan Wang is at https://github.com/hwang0113/Prognostic-System-for-Lymphoma (accessed on 29 May 2025). The source code was modified in R (Version 4.4.2) to generate the results in this manuscript.

## Abbreviations

AJCC	American Joint Committee on Cancer
C-index	Concordance Index
TNM	Tumor, Lymph Node, and Metastasis
EACCD	Ensemble Algorithm for Clustering Cancer Data
SEER	Surveillance, Epidemiology, and End Results
pNETs	pancreatic neuroendocrine tumors
ENETS	European Neuroendocrine Tumor Society
AI	Artificial Intelligence
